# Genetic signatures of *ERCC1* and *ERCC2* expression, along with SNPs variants, unveil favorable prognosis in SCLC patients undergoing platinum-based chemotherapy

**DOI:** 10.32604/or.2024.050161

**Published:** 2024-12-20

**Authors:** ENRICO CALIMAN, SARA FANCELLI, FEDERICO SCOLARI, ADRIANO PASQUI, CLARA MANNESCHI, DANIELE LAVACCHI, FRANCESCA MAZZONI, FRANCESCA GENSINI, VALERIA PASINI, CAMILLA EVA COMIN, LUCA VOLTOLINI, SERENA PILLOZZI, LORENZO ANTONUZZO

**Affiliations:** 1 Clinical Oncology Unit, Careggi University Hospital, Florence, 50134, Italy; 2 Department of Experimental and Clinical Medicine, University of Florence, Florence, 50134, Italy; 3 Department of Health Sciences, University of Florence, Florence, 50134, Italy; 4 Medical Oncology Unit, Careggi University Hospital, Florence, 50134, Italy; 5 Department of Experimental and Clinical Biomedical Sciences “Mario Serio”, University of Florence, Florence, 50134, Italy; 6 Section of Anatomic Pathology, Department of Health Sciences, University of Florence, Florence, 50139, Italy; 7 Section of Surgery, Histopathology and Molecular Pathology, University of Florence, Florence, 50139, Italy; 8 Thoracic Surgery Unit, Careggi University Hospital, Florence, 50134, Italy

**Keywords:** Small cell lung cancer (SCLC), Nucleotide excision repair (NER) pathway, *ERCC* genes, Single nucleotide polymorphisms (SNPs), Platinum-chemotherapy (CT)

## Abstract

**Background:**

Platinum chemotherapy (CT) remains the backbone of systemic therapy for patients with small-cell lung cancer (SCLC). The nucleotide excision repair (NER) pathway plays a central role in the repair of the DNA damage exerted by platinum agents. Alteration in this repair mechanism may affect patients’ survival.

**Materials and Methods:**

We conducted a retrospective analysis of data from 38 patients with extensive disease (ED)-SCLC who underwent platinum-CT at the Clinical Oncology Unit, Careggi University Hospital, Florence (Italy), from 2015 to 2020. mRNA expression analysis and single nucleotide polymorphism (SNP) characterization of three NER pathway genes—namely *ERCC1*, *ERCC2*, and *ERCC5*—were performed on patient tumor samples.

**Results:**

Overall, elevated expression of *ERCC* genes was observed in SCLC patients compared to healthy controls. Patients with low *ERCC1* and *ERCC5* expression levels exhibited a better median progression-free survival (mPFS = 7.1 *vs*. 4.9 months, *p* = 0.39 for *ERCC1* and mPFS = 6.9 *vs*. 4.8 months, *p* = 0.093 for *ERCC5*) and overall survival (mOS = 8.7 *vs*. 6.0 months, *p* = 0.4 for *ERCC1* and mOS = 7.2 *vs*. 6.2 months, *p* = 0.13 for *ERCC5*). Genotyping analysis of five SNPs of *ERCC* genes showed a longer survival in patients harboring the wild-type genotype or the heterozygous variant of the *ERCC1* rs11615 SNP (*p* = 0.24 for PFS and *p* = 0.14 for OS) and of the rs13181 and rs1799793 *ERCC2* SNPs (*p* = 0.43 and *p* = 0.26 for PFS and *p* = 0.21 and *p* = 0.16 for OS, respectively) compared to patients with homozygous mutant genotypes.

**Conclusions:**

The comprehensive analysis of *ERCC* gene expression and SNP variants appears to identify patients who derive greater survival benefits from platinum-CT.

## Introduction

Small-cell lung cancer (SCLC) accounts for approximately 10%–20% of primary lung tumors and most patients present with extensive disease (ED) at the time of diagnosis. Despite a high rate of response to first-line treatment, responses are transient, and patients often experience a rapid disease relapse resulting in poor 2-year survival outcomes [1]. Recently, the introduction of immune checkpoint inhibitors (ICIs) in combination with platinum-based chemotherapy (CT) has set a new standard of care for the treatment of ED-SCLC. Nonetheless, platinum-based CT remains the backbone of first-line systemic therapy for these patients [2–4].

The dismal prognosis of SCLC is strongly related to the development of CT resistance mechanisms by the tumor, including those based on DNA damage repair systems. Platinum agents, particularly cisplatin and carboplatin, exert their cytotoxic effects by inducing DNA damage through the formation of intrastrand adducts and interstrand cross-links that inhibit DNA synthesis and transcription [5]. Nucleotide excision repair (NER) pathway plays a central role among DNA damage repair systems and is strictly involved in the detection and repair of DNA adducts and cross-links induced by platinum activity [6,7]. The NER pathway consists of several steps: recognition, incision/excision of DNA lesion, restoration through the synthesis of new nucleotides, and ligation [8]. Over 30 different proteins participate in this process and converge at the DNA repair site into the TFIIH transcription initiation complex [9]. Proteins that play a crucial role in this pathway, including excision repair cross-complementing group 1 (*ERCC1*), *ERCC2*, and *ERCC5* have been widely studied in different types of tumors, including lung cancer [10]. Different expression levels of these proteins can alter DNA repair capacity, modulate cancer susceptibility, and, eventually, impact therapy response and survival of lung cancer patients treated with platinum compounds [11]. Moreover, several studies have shown that genetic factors such as single nucleotide polymorphisms (SNPs) in specific genes of the NER pathway may influence the inter-individual differences in platinum effectiveness and toxicity [12,13]. However, the results still remain controversial. To date, far fewer studies have investigated the prognostic and predictive role of these NER pathway genes in patients with SCLC, with no definitive evidence of their influence on platinum-CT responsiveness or patient survival [14–17].

The aim of our study was to determine the correlation between these genes of the NER pathway and survival outcomes in patients with SCLC. To this aim, we retrospectively analyzed the *ERCC1*, *ERCC2*, and *ERCC5* expression levels and the genotypic characterization of a panel of five single nucleotide polymorphisms (SNPs) of these genes in a cohort of patients with ED-SCLC who received platinum/etoposide regimens.

## Materials and Methods

### Patients

In this single-center observational study, we retrospectively collected clinical-demographic data from medical records of patients with ED-SCLC (according to the VII edition of TNM staging system), who were treated at the Clinical Oncology Unit, Careggi University Hospital, Florence (Italy) between January 2015 to December 2020. All patients enrolled in the analysis received platinum-based standard of care CT as first-line treatment, which was administered up to a maximum of six cycles or was discontinued earlier due to disease progression, intolerable toxicity, clinical decision, or patient refusal. Patients’ responses to CT were assessed with radiological imaging every three months, according to clinical practice. The radiological complete response (CR), partial response (PR), stable disease (SD), and progression disease (PD) were defined in accordance with the Response Evaluation Criteria in Solid Tumors (RECIST) version 1.1. The measured clinical outcomes were the following: Objective Response Rate (ORR, defined as CR plus PR) and Disease Control Rate (DCR, defined as the percentage of patients who have achieved CR or PR or SD), the Progression-Free Survival (PFS) and the Overall Survival (OS). The cut-off date for follow-up analysis was 31 December 2021.

### Data collection

The following characteristics were reviewed from each patient’s medical record: a) clinical-demographic data, i.e., age, sex, Eastern Cooperative Oncology Group Performance Status (ECOG PS), smoking status, and metastasis sites at diagnosis; b) histopathological data from Formalin-Fixed Paraffin-Embedded (FFPE) archival diagnostic tumor tissues of the study population, obtained in collaboration with the Histopathology and Molecular Pathology Unit, Careggi University Hospital, Florence) data about treatment received by patients, such as the type of chemotherapy administered in the first and subsequent lines, the duration of each line of therapy, the radiological responses obtained, and the type of radiotherapy performed, if any (symptomatic or prophylactic cranial irradiation, PCI). Archival FFPE tumor samples of patients were collected and further analyzed for mRNA expression and genotype characterization of key genes of the NER pathway, namely *ERCC1*, *ERCC2*, and *ERCC5*. In detail, mRNA expression was available from a cohort of n = 26 samples, while the analysis of a panel of five SNPs of the genes was assessed in the entire study population (n = 38). Four lung tissue samples from healthy donors were used as a control group for the gene expression analysis.

### DNA extraction and genotyping by SNP assay

DNA was extracted from SCLC FFPE sections using a QIAamp DNA Blood mini kit (Qiagen, Manchester, UK). Allelic discrimination was performed using TaqMan SNP Genotyping Assays (Applied Biosystems, Forster City, CA, USA) on the Rotor-gene 6000 instrument (Qiagen, Manchester, UK). The assay consisted of two allele-specific minor groove binding (MGB) probes, labeled with the fluorescent dyes VIC and FAM. Real-time PCR was performed in 15 μL reaction mixtures containing 7.5 μL of Taqpath ProAmp MasterMix (Applied Biosystems, Forster City, CA, USA). TaqMan-MGB genotyping assay mixes were supplied at 40X concentration and 2 μL of sample DNA. Thermocycler conditions were an initial hold step for 30 s at 60°C and 5 min at 95°C, followed by 40 cycles of 95°C for 15 s and 60°C for 60 s, and finally, a last post read step for 30 s at 60°C. Genotypes were analyzed by measuring allele-specific fluorescence using the Rotor-gene software for allelic discrimination (Qiagen, Manchester, UK).

### RNA extraction and quantitative real-time reverse transcription PCR (qPCR)

Total RNA was extracted from SCLC FFPE sections using the RNeasy Mini kit (Qiagen, Manchester, UK). The quantity and the quality of RNA were evaluated using a Nanodrop spectrophotometer. 50 ng of RNA from each sample were retro-transcribed with the PrimeScript™ RT Reagent Kit (Takara Bio, Japan); the resulting cDNA was then used for qPCR analysis with “Powerup Sybr Green” (Applied Biosystems, Forster City, CA, USA) on a Rotor Gene Q (Qiagen, Manchester, UK) using *ERCC1* (F 5′CTCAAGGAGCTGGCTAAGATGT3′; R 5′CATAGGCCTTGTAGGTCTCCAG3′), *ERCC2* (F 5′CTGGAGGTGACCAAACTCATCTA3′ R 5′CCTGCTTCTCATAGAAGTTGAGC3′) and *ERCC5* (F 5′CAGACACAGCTCCGAATTGA3′ 5′TTCTGGGTTTTTCGTTTTGC3′) primers. The relative quantification was performed using LinRegPCR software and the data were normalized to 18s rRNA. The primers used are listed below.

### Statistical analysis

Patient characteristics were presented by descriptive statistics. Statistical analysis was performed using R [18]. The frequency distribution of the five SNPs in our cohort and the general population has been compared by chi-squared test. Relative expression levels have been obtained with the 2^−∆∆Ct^ method [19], using as reference gene 18S RNA and as negative control the geometric mean of the expression levels in 4 healthy tissue samples. The correlation between *ERCC* genes’ relative expression and clinical or genotypic parameters has been computed by the Wilcoxon test. Multiple testing corrections have been performed with the Bonferroni method. For each of the three evaluated genes, the cutoff point for “high” and “low” expression levels was estimated by maximizing the Youden Index of the Receiver Operating Characteristic (ROC) curve. The ROC curve was plotted using as parameters the gene expression of each *ERCC* gene and the 12 months overall survival. This analysis has been performed with the ROCR package [20]. Kaplan-Meier curves have been computed with the package for survival analysis in R, illustrating the correlation between expression levels or SNP variants and OS or PFS. Linkage Disequilibrium (LD) analysis was performed and plotted using the Gaston package for R.

### Ethics approval

The present study was approved by the Regional Ethics Committee for Clinical Trials of the Tuscany Region (“QUASAR” protocol, code “19822_BIO”). All informed consent documents are in compliance with the International Conference on Harmonization (ICH) guideline on good clinical practice (GCP). The study protocol is performed in accordance with the principles of the Declaration of Helsinki and in compliance with GCP and the applicable laws and regulations. Each patient will be identified by a code instead of the patient’s name in order to protect the patient’s identity when reporting study-related data.

## Results

### Patient characteristics

Thirty-eight patients with advanced or relapsed SCLC who received CT as per clinical practice during the study period were included in the analysis. The clinical baseline characteristics of the enrolled patients are summarized in Table 1. The median age at the time of diagnosis was 68 years (range 41–81 years), 17 were males (44.74%) and 21 were females (55.26%). All patients had a history of tobacco exposure (n = 14 former and n = 24 current smokers). At enrolling time, the ECOG PS was 1 in half of the patients (n = 19, 50.00%), 0 in 11 patients (28.95%), and 2 in 8 patients (21.05%). All patients had ES-SCLC and received platinum-based CT as frontline therapy. Carboplatin plus etoposide was used as first-line treatment in 34 patients (89.47%), while 4 (10.53%) patients were treated with cisplatin plus etoposide. Most patients received 4 up to 6 cycles of CT (n = 26, 68.42%), while 12 patients (31.58%) received 3 or fewer cycles. The most frequent metastasis sites at diagnosis were respectively: liver (n = 18, 47.37%), bone (n = 18, 47.37%), adrenal glands (n = 15, 39.47%), and brain (n = 7, 18.42%).

**Table 1:** Patients’ baseline characteristics

Patients (n = 38)	
Age	
Median (range)–years	68 (41–81)
Sex–no. (%)	
Male	17 (44.74)
Female	21 (55.26)
Smoking status–no. (%)	
Never	0 (0)
Current	24 (63.16)
Former	14 (36.84)
ECOG performance status–no. (%)	
0	11 (28.95)
1	19 (50.00)
2	8 (21.05)
Metastasis sites–no. (%)	
Liver	18 (47.37)
Bone	18 (47.37)
Adrenal gland	15 (39.47)
Brain	7 (18.42)
Treatments	
I line–no. (%)	38 (100)
Carboplatin plus etoposide	34 (89.47)
Cisplatin plus etoposide	4 (10.53)
Number of cycles	
≤3	12 (31.58)
4–6	26 (68.42)
II line–no. (%)	8 (21.05)
III line–no. (%)	3 (7.89)
Best response to treatment–no. (%)	
Complete response	1 (2.63)
Partial response	21 (56.26)
Stable disease	1 (2.63)
Progression disease	15 (39.47)
Radiotherapy–no. (%)	
Chest/mediastin	7 (18.42)
Bone	4 (10.53)
Brain	4 (10.53)
Liver	0 (0)
Adrenal gland	0 (0)
PCI–no. (%)	5 (13.16)

In the overall population, 1 patient achieved CR (2.63%), 21 PR (56.26%), and 1 SD (2.63%), while 15 had PD as best response (39.47%). All patients experienced PD or death at the cut-off date for the follow-up analysis. Preferred sites of disease recurrence were the chest, liver, bone, brain, or adrenal glands. Eight patients (21.05%) received a second-line CT with topotecan (n = 1, 2.63%) or irinotecan (n = 4, 10.53%), or platinum plus etoposide (n = 3, 7.89%) or other (n = 1, 2.63%), while only 3 patients (7.89%) were fit for a third line. Fifteen patients (39.47%) underwent palliative-symptomatic radiotherapy to the chest, bone, or brain, while in 5 patients (13.16%), who achieved an excellent response rate to first-line CT, prophylactic cranial irradiation (PCI) was performed.

### Expression of NER pathway genes and their association with clinicopathological characteristics

**Figure 1:**
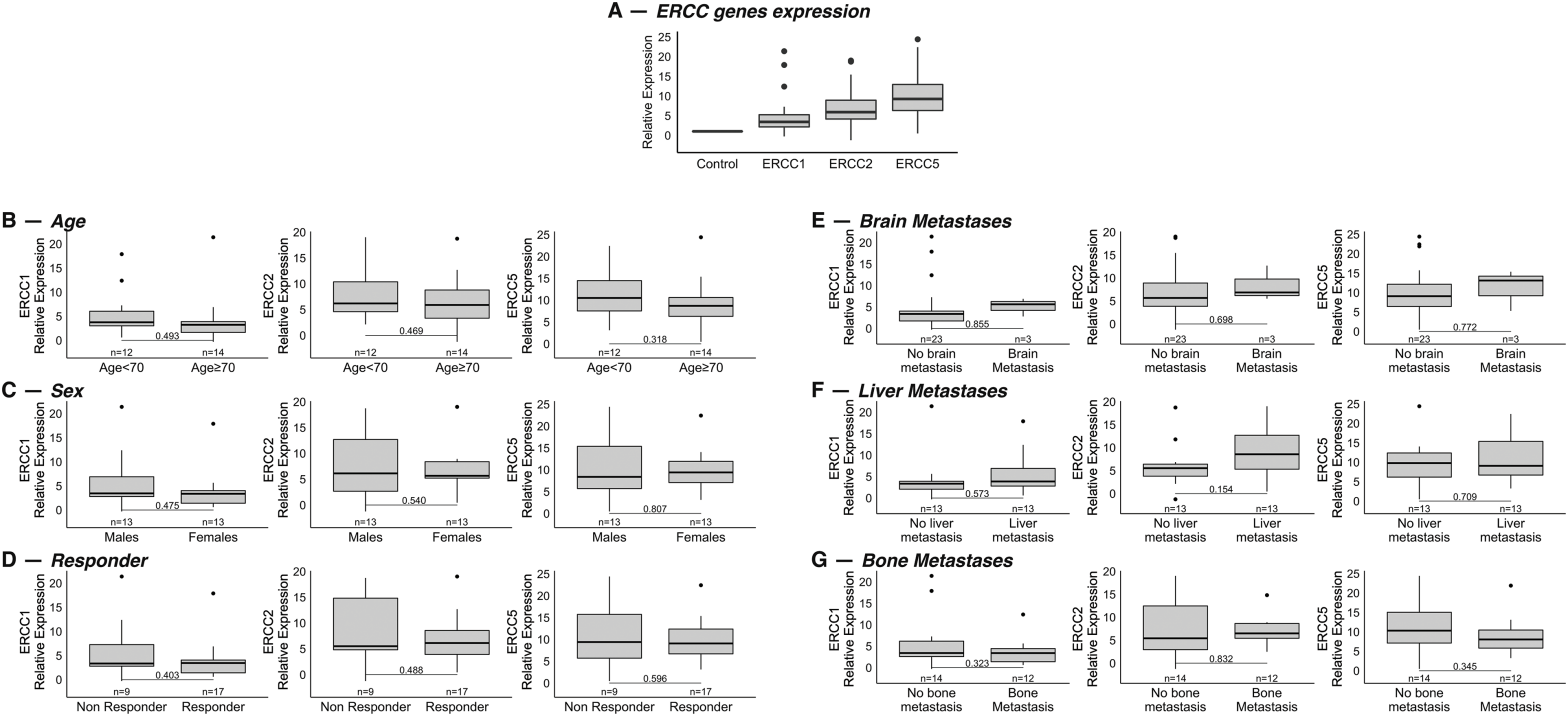
(A): Relative mRNA expression values of the genes *ERCC1*, *ERCC2* and *ERCC5* in a cohort of 26 patients with extended stage-SCLC compared to the healthy group. Values were normalized to the control group, value = 1. (B–G): Association between mRNA expression levels of the genes and age (< or ≥70 years) (B) (*p* = 0.49 for *ERCC1, p* = 0.47 for *ERCC2*, *p* = 0.32 for *ERCC5*), sex (C) (*p* = 0.47 for *ERCC1, p* = 0.54 for *ERCC2*, *p* = 0.81 for *ERCC5*), response to therapy (D) (*p* = 0.40 for *ERCC1, p* = 0.49 for *ERCC2*, *p* = 0.60 for *ERCC5*) and site of metastasis at diagnosis: brain (E) (*p* = 0.85 for *ERCC1, p* = 0.70 for *ERCC2*, *p* = 0.77 for *ERCC5*), liver (F) (*p* = 0.57 for *ERCC1, p* = 015 for *ERCC2*, *p* = 0.71 for *ERCC5*) and bone (G) (*p* = 0.32 for *ERCC1, p* = 0.83 for *ERCC2*, *p* = 0.34 for *ERCC5*).

The expression levels of the three genes of interest involved in the NER pathway (i.e., *ERCC1*, *ERCC2*, and *ERCC5*) were obtained from diagnostic FFPE tumor samples. Tumor samples showed higher levels of gene expression than healthy controls: in detail, the median expression values of the three genes in the study population compared to the control group were 3.41 (95% Confidence Interval [CI], 0.27–19.20) for *ERCC1*, 5.89 (95% CI, 0.19–18.84) for *ERCC2* and 9.24 (95% CI, 2.12–23.17) for *ERCC5*, respectively (Fig. 1A).

### SNPs analysis

We then genotyped five polymorphic alterations in a panel of SNPs belonging to relevant genes of the NER pathway: rs11615 for *ERCC1*, rs13181 and rs1799793 for *ERCC2*, rs2296147 and rs1047768 for *ERCC5*. Data was available for all patients enrolled in the study (n = 38). Genotyping of rs11615 in the *ERCC1* gene indicated that the most common genotype was A/G (42%; 16/38), while 53% (20/38) and 58% (22/38) of patients showed T/G genotype in rs13181 and C/T genotype of rs1799793 in the *ERCC2* gene, and 50% (19/38) and 47% (18/38) of patients showed T/C genotype in rs2296147 and T/C or CC genotype of rs1047768 in the *ERCC5* gene (Frequencies of genotypes are shown in Table S1). Linkage disequilibrium (LD) coefficient r2 was calculated to evaluate the non-random association of *ERCC* SNPs alleles at different loci. A moderate LD was observed between; i) *ERCC2* SNPs rs13181 and re1799793; ii) *ERCC5* SNPs rs1047768 and rs2296147; iii) *ERCC1* SNP rs11615 and *ERCC2* SNP rs1799793 (Fig. S1).

As far as the correlation between SNPs variants and clinicopathological characteristics of patients, our results showed no significant association between SNPs and patient features (data not shown).

In the 26 patients for whom mRNA expression values were available, we assessed whether they correlated with the main clinicopathological features of the study population, such as age (< or ≥70 years), sex, site of metastasis, and response to therapy (responder *vs*. no responder). No statistically significant association was found between gene expression levels and age, sex, or response to treatment (Fig. 1B–D). With regard to the association with the site of metastasis, results showed no statistical correlation between gene expression and different sites of metastasis (Fig. 1E–G).

### Association between SNPs variants and gene expression

**Figure 2:**
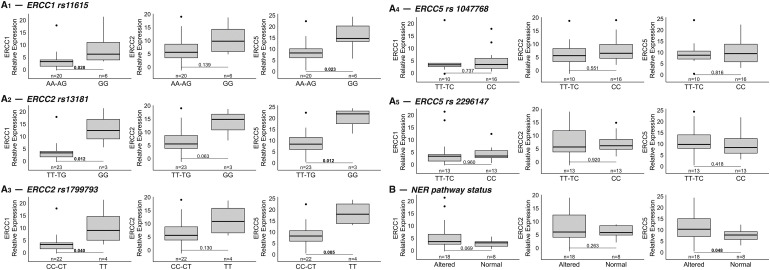
(A) Correlation between SNPs variants of the three genes and their expression. Five SNPs of the genes of interest were analyzed: rs11615 for *ERCC1* (top row on the left: 2A_1_), rs13181 and rs1799793 for *ERCC2* (second and third rows on the left: 2A_2_ and 2A_3_), rs2296147 and rs1047768 for *ERCC5* (first and second rows on the right: 2A_4_ and 2A_5_). Mutant homozygous variants of *ERCC1* and *ERCC2* SNPs (right of each boxplot) were statistically associated with increased expression of all three genes compared to wild-type homozygous and alternative heterozygous variants (left of each boxplot). Different gene expression patterns were found for variants of the two *ERCC5* SNPs. The data refer to the 26 patients for whom the mRNA expression levels of each gene were available. (B) Association between NER pathway deficiency and gene expression. Deficiency of the NER pathway correlates with higher gene expression levels of *ERCC1*, *ERCC2* and *ERCC5* (NER pathway was considered deficient [“altered”] if at least one of the three genes had a mutant homozygous variant in one of the five SNPs of interest). Note: The corresponding *p*-values are indicated in each figure for ease of reference.

We next analyzed the correlation between SNPs variants of the three genes and their expression: patients harboring mutant homozygous variants of the *ERCC1* rs11615 and of the two SNPs of *ERCC2* (rs13181 and rs1799793) had significantly higher expression of the corresponding gene (Fig. 2A). Of note, patients with the mutant homozygous variant of the *ERCC1* SNP also had higher expressions of the other two genes, *ERCC2* and *ERCC5*. Similarly, patients harboring the mutant homozygous variants of the two SNPs of *ERCC2* showed increased gene expression of *ERCC1* and *ERCC5*. However, the mutant homozygous variants of the two SNPs of *ERCC5* (rs1047768 and rs2296147) were associated with a different gene expression pattern (Fig. 2A). We then assessed whether alteration of the NER pathway by the presence of variants in the SNPs was associated with different expression levels of the three genes. In detail, patients were considered deficient in the NER pathway if at least one of the three genes harbored a mutant homozygous variant in one of the five SNPs. Consistently with previous results, a positive association was found between NER pathway deficiency and higher gene expression levels of *ERCC1* (*p* = 0.069), *ERCC2* (*p* = 0.26) and *ERCC5* (*p* = 0.048) (Fig. 2B).

### Survival outcomes analysis

**Figure 3:**
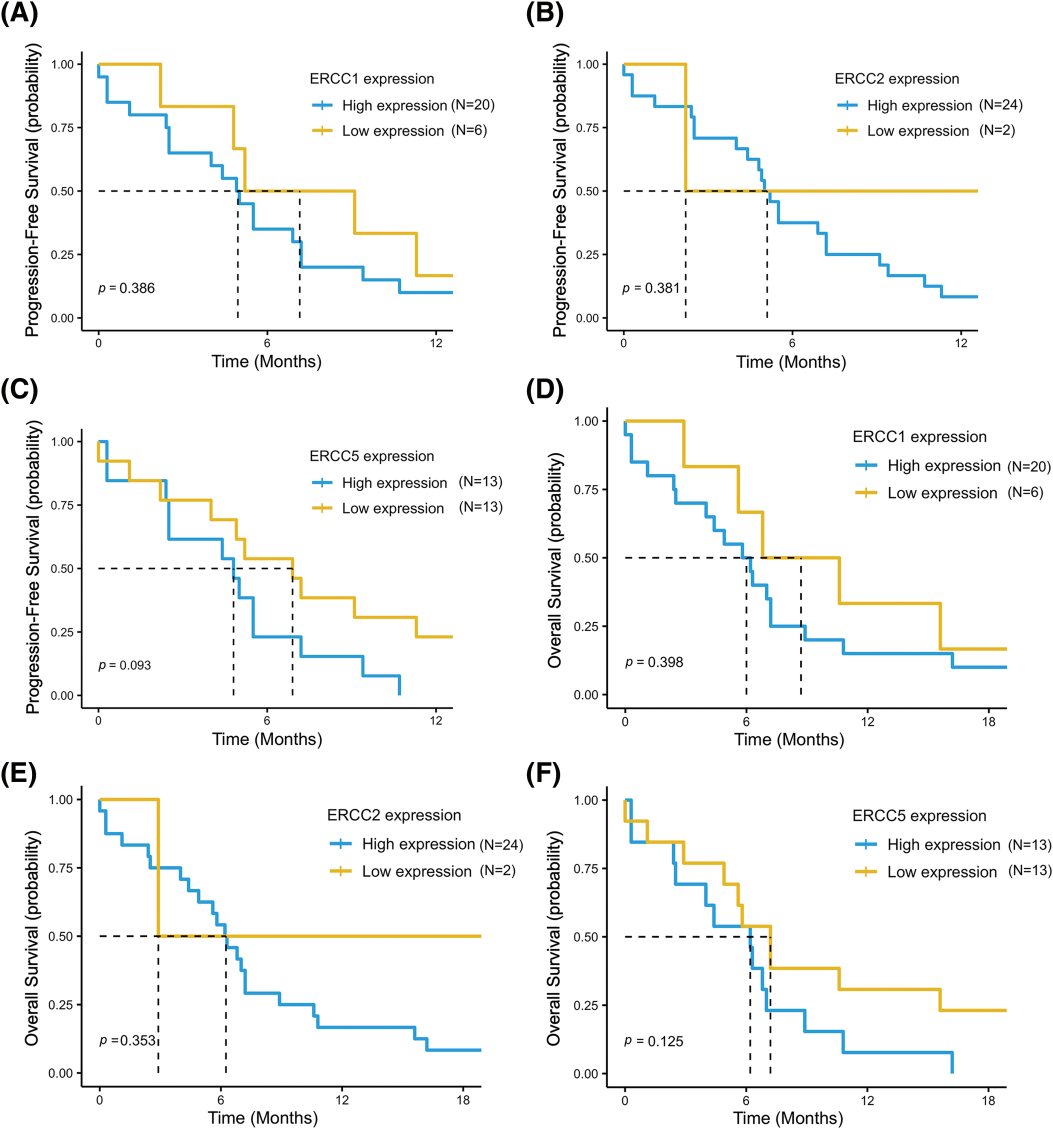
Progression free survival (PFS) and overall survival (OS) in the low- *vs*. high-expression groups for each of the three genes: *ERCC1* (A and D), *ERCC2* (B and E) and *ERCC5* (C and F). The data refer to the 26 patients for whom the mRNA expression levels of each gene were available.

In the overall population, our results showed a median PFS (mPFS) of 4.5 months (95% CI, 4.0–7.2) and a mOS of 5.7 months (95% CI, 4.4–7.2). For the analysis of survival outcomes, the study population was divided into high and low expression groups for each of the three genes, based on the cutoff point estimated by ROC curve analysis. Patients with low *ERCC1* and *ERCC5* expression showed a longer PFS compared to patients with high expression (mPFS = 7.1 months (95% CI, 4.8–NR) *vs*. 4.9 months (95% CI, 2.5–7.2) *p* = 0.39, for *ERCC1* and mPFS = 6.9 months (95% CI, 4.0–NR) *vs*. 4.8 months (95% CI, 2.5–NR) *p* = 0.093, for *ERCC5*) (Fig. 3A,C).

Conversely, patients with low *ERCC2* expression had lower PFS than patients with high expression (mPFS = 2.2 months (95% CI, 2.2–NR) *vs*. 5.1 months (95% CI, 4.4–7.2) in low and high expression groups, respectively, *p* = 0.38) (Fig. 3B). Consistently, a trend for a better OS was reported in the low expression groups for *ERCC1* (mOS = 8.7 months (95% CI, 5.6–NR) *vs*. 6.0 months (95% CI, 4.0–8.9), *p* = 0.4) and for *ERCC5* (mOS = 7.2 months (95% CI, 4.9–NR) *vs*. 6.2 months (95% CI, 2.5–NR), *p* = 0.13) and in the high expression group for *ERCC2* (mOS = 6.2 months (95% CI, 4.9–8.9) *vs*. 2.9 months (95% CI, 2.9–NR), *p* = 0.35) (Fig. 3D–F).

**Figure 4:**
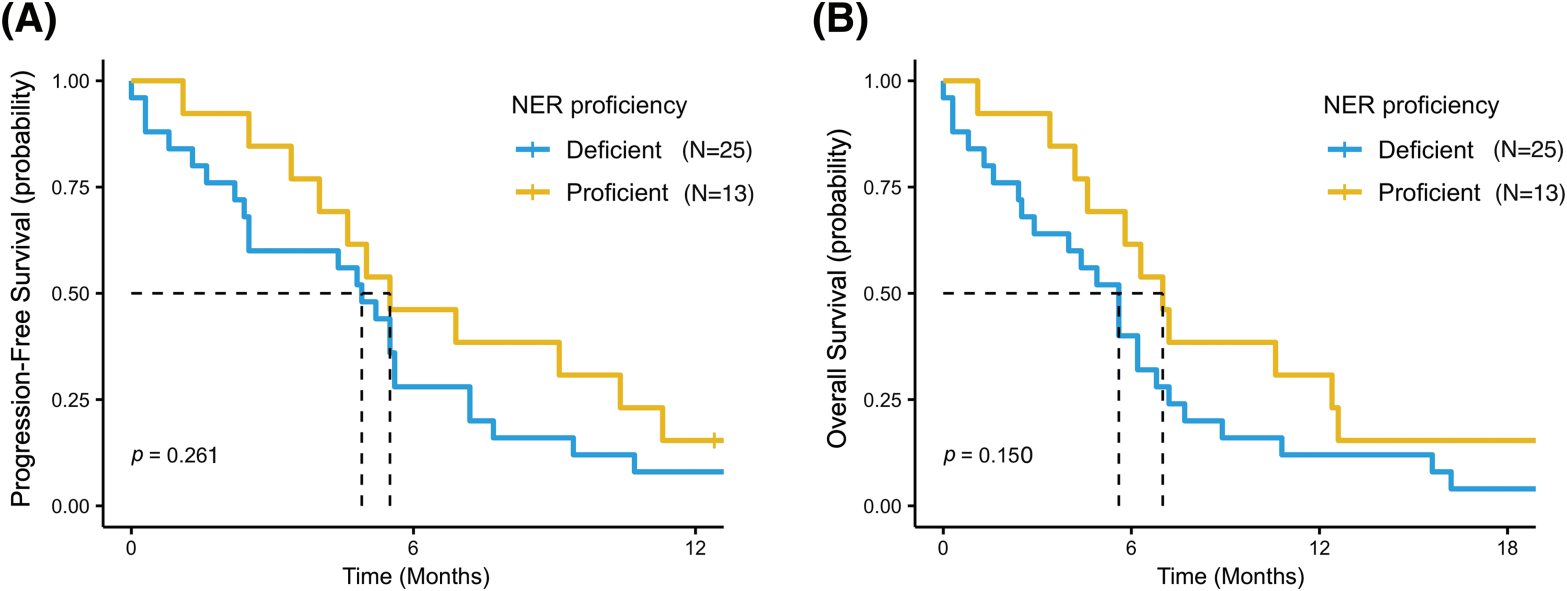
Progression free survival (PFS) (A) and overall survival (OS) (B) in patients with wild-type or heterozygous genotypes (NER pathway “proficient”) and in patients with homozygous mutant variant in at least one of the five SNPs (NER pathway “deficient”).

Survival outcomes analysis was also performed according to SNPs variants in the entire study population. Overall, better PFS and OS were reported in patients with wild-type or heterozygous genotypes (NER pathway “proficient”) than in patients with homozygous mutant variants (NER pathway “deficient”): mPFS = 5.5 months (95% CI, 4.0–NR) *vs*. 4.9 months (95% CI, 2.5–7.2) (*p* = 0.26) and mOS = 7.0 months (95% CI, 4.6–NR) *vs*. 5.6 months (95% CI, 2.9–7.2) (*p* = 0.15), respectively (Fig. 4A,B). Accordingly, a non-significant trend for a better PFS and OS was reported for patients with a wild-type genotype or heterozygous variant than for patients with a homozygous mutant variant for all SNPs examined (Table S2).

**Figure 5:**
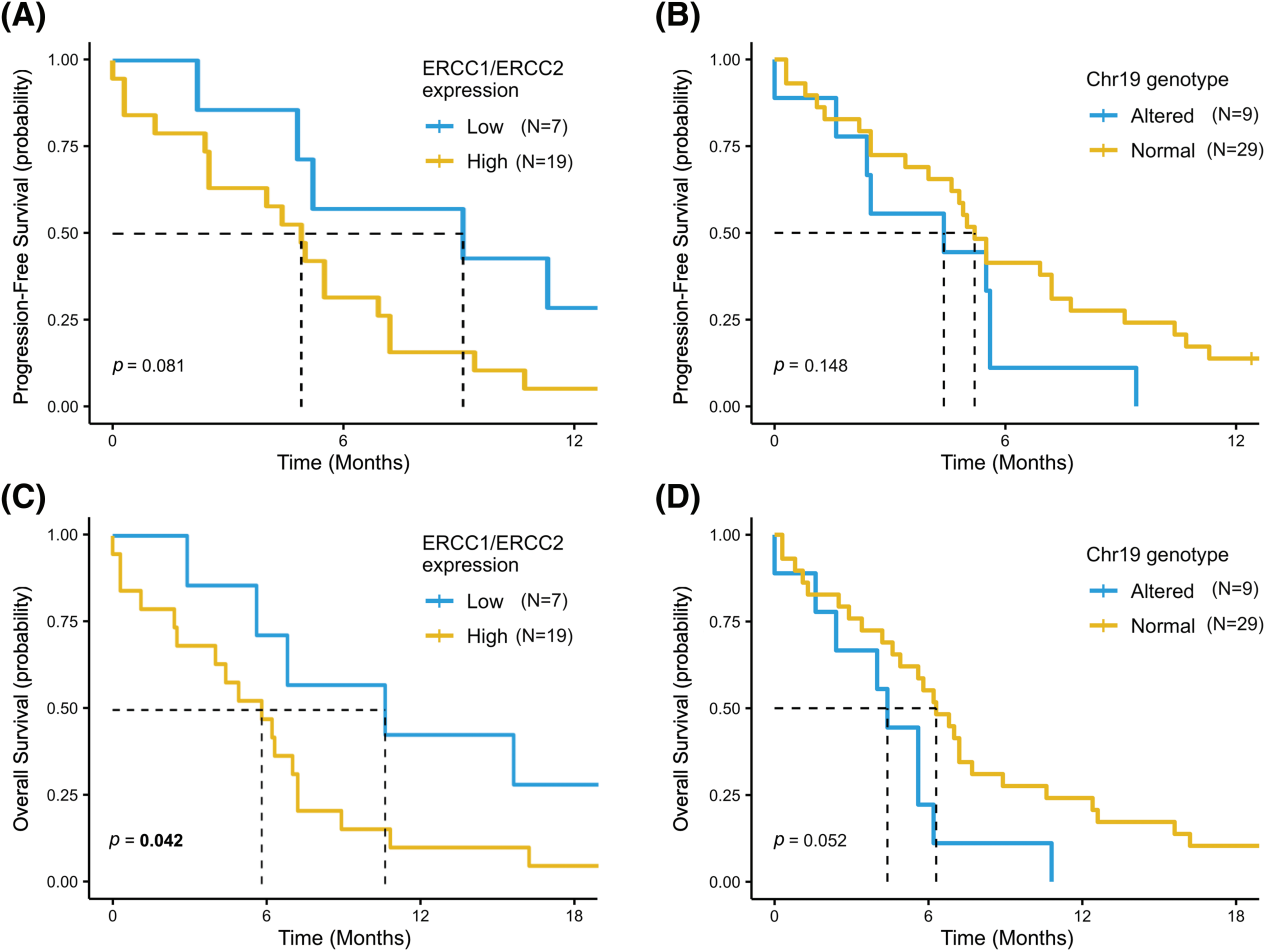
Progression Free Survival (PFS) and Overall Survival (OS) according to *ERCC1* and *ERCC2* status. PFS and OS was assessed in patients with low *ERCC1* or *ERCC2* expression compared to high-expression subgroup (A and B) and in patients with at least one mutant homozygous variant in *ERCC1* or *ERCC2* SNPs *vs.* patients with wild-type or heterozygous SNPs variant (C and D).

Finally, as *ERCC1* and *ERCC2* genes are both located in chromosome 19 and the linkage disequilibrium analysis showed a moderate association of their SNPs alleles, we assessed the survival outcomes of patients based on gene expression together with SNPs variants of the two genes. As shown in Fig. 5, patients with low *ERCC1* or *ERCC2* expression showed a statistically significantly better OS than patients with high gene expression (mOS = 10.6 months [95% CI, 5.6–NR] *vs*. 5.6 months (95% CI, 4.0–7.2), *p* = 0.042) (Fig. 5C). Similarly, patients with wild-type or heterozygous SNPs variants in *ERCC1* or *ERCC2* SNPs had a mOS of 6.3 months (95% CI, 4.9–8.9), compared to mOS of 4.4 months (95% CI, 2.4–NR) in patients with at least one mutant homozygous variant (*p* = 0.052) (Fig. 5D). A similar trend, although not statistically significant, was also reported for PFS in these two patient subgroups (Fig. 5A,B).

## Discussion

The NER pathway is one of the major repair mechanisms for platinum-DNA adducts and cross-links, playing a key role in the detection and repair process of the DNA damage exerted by the platinum compounds, particularly cisplatin and carboplatin [6,7]. Several studies have described those genetic alterations in the SNPs of NER pathway genes and deregulation of NER pathway proteins may affect the DNA repair functions, influence the efficacy of platinum-CT, and, eventually, impact on clinical outcomes of patients with lung cancer [10,21].

In our study we investigated a homogeneous cohort of 38 patients with ED-SCLC, assessing the role of three key genes encoding crucial factors of the NER pathway, *ERCC1*, *ERCC2*, and *ERCC5*. Our results showed a higher expression of these genes in SCLC patients compared to the healthy controls; of note, patients with low *ERCC1* and *ERCC5* expression had better mPFS and mOS, while an inverse trend emerged in survival outcomes for *ERCC2* expression. As SNPs can influence gene functions and phenotype, potentially leading to increased mRNA expression [22], genotyping of *ERCC* genes was conducted to investigate how various *ERCC* SNP profiles might correlate with altered expression levels. Specifically, the analysis examined five polymorphic alterations within a panel of clinically relevant SNPs associated with these genes. The results revealed that patients with homozygous mutant genotypes in the *ERCC1* rs11615 SNP and both the *ERCC2* rs13181 and rs1799793 SNPs exhibited heightened expression of all three genes. Conversely, a distinct gene expression pattern emerged for the genetic variants of the two *ERCC5* SNPs (rs1047768 and rs2296147). These findings are consistent with similar results previously reported by our group [23]. Finally, we investigated how genetic alterations in these SNPs may influence survival outcomes of SCLC patients. Overall, our data showed a longer PFS and OS, although not significant, in patients harboring the wild-type genotype or the heterozygous variant of the *ERCC1* rs11615 SNP and of the rs13181 and rs1799793 *ERCC2* SNPs compared to patients with the corresponding homozygous mutant genotype. On the other hand, the two polymorphisms of *ERCC5* showed no association with patients’ survival.

The higher gene expression of *ERCC1*, *ERCC2*, and *ERCC5* in tumor samples compared to healthy control is consistent with previous studies that reported high tumor tissue levels of NER pathways genes, mainly *ERCC1*, in SCLC and in other solid tumors [14,15,24,25]. The high expression of NER pathway genes could lead to an increased ability of tumor cells to repair the DNA damage exerted by platinum-CT, conferring resistance to anti-cancer treatment. This may explain why, despite initial response to therapy, almost all ED-SCLC patients eventually experience disease progression and often early disease relapse. Consistent with this observation, *ERCC1* has been extensively investigated as a predictive biomarker for the efficacy of platinum agents and as a negative prognostic factor in various malignancies, including NSCLC and SCLC. Several studies have shown a significant correlation between low *ERCC1* expression and both higher response rates to CT and better clinical outcomes in cancer patients [11,26–29]. As far as SCLC, protein or mRNA expression of *ERCC1* was reported to be predictive of treatment efficacy and a prognostic factor for survival [14,30–32]. Moreover, in a large retrospective study of 184 SCLC patients, the low expression of *ERCC1*, as part of a favorable expression signature, was significantly correlated with better PFS and OS in both limited disease (LD) and ED-SCLC [16]. Interestingly, Lee et al. also reported that expression of *ERCC1* in SCLC is lower compared to studies of tissue from NSCLC, suggesting that the greater biological aggressiveness of SCLC could be related to the loss of *ERCC1* gene, which leads to impaired DNA-repair functions of the NER pathway [33]. According to previous data, our findings showed that patients with low *ERCC1* expression had longer PFS and OS. Similarly, better outcomes were also found in patients with low *ERCC5*, for whom far less data is available to date [34,35]. Of note, Simon and colleagues reported that increased expression of *ERCC1* is an independent predictor of improved survival in resected patients with NSCLC and that this may be secondary to a decreased accumulation of genomic aberrations as a result of efficient DNA-damage repair system [36]. The favorable prognostic value of *ERCC1* expression in resected-NSCLC patients was also confirmed by Olaussen et al. [37]. Consequently, upregulation of *ERCC1* on the one hand seems to be a negative prognostic factor in advanced disease, as it reduces the benefit of platinum-CT, while on the other hand may act as a predictor of better survival in limited disease, as it reduces the risk of relapse after definitive treatment. Interestingly, it was reported that the *ERCC1* gene generates different isoforms by alternative splicing and that only one isoform is implicated in the repair of platinum-DNA adducts [38]. Consequently, the expression of different isoforms could be a major concern as it would lead to a tumor being considered *ERCC1*-positive, although the expressed protein might be non-functional.

In contrast with *ERCC1* and *ERCC5*, our results showed that patients with low *ERCC2* expression had an opposite survival trend with shorter PFS and OS. Although this finding should be taken with caution given the small size of *ERCC2*-low subgroup in our population, some previous data have reported a correlation between *ERCC2* upregulation with a more aggressive cancer phenotype in head and neck tumors and in NSCLC cell lines, suggesting an inter-tissue variation in NER genes and chemoresistance [39–41].

The genotyping analysis of SNPs on NER genes in our cohorts showed that the allele frequencies of the three genes of interest differed between SCLC patients and the general population, particularly for the SNP of *ERCC5* rs2296147 (*p* < 0.0001), suggesting a crucial role of the NER pathway in these patients. As wild-type and variant genotypes of the NER pathway are likely associated with differential activity of DNA repair functions [42], polymorphic alterations in these genes may influence the variability of DNA damage repair activity. Consequently, we assessed whether specific genotypes of these genes may also impact the clinical outcomes of SCLC patients. Our data showed that homozygous mutant genotypes in the SNPs of *ERCC1* and *ERCC2* are associated with decreased PFS and OS compared to wild-type or heterozygous variant genotypes. To date, very few studies have investigated the role of genetic polymorphisms in the NER pathway for SCLC. Nicos and colleagues evaluated the genotypes of the *ERCC1* in a cohort of SCLC patients, reporting that patients harboring the heterozygous genotype in rs11615 had a significantly shorter OS compared to the wild-type genotype, which instead was a favorable prognostic factor. Moreover, the different genotypes in two SNPs of the *ERCC1* showed also a correlation with the hematological toxicity of the treatment [17]. Conversely much more data about the role of gene polymorphisms in the NER pathway are available for NSCLC, where they have been extensively investigated, sometimes with contradictory results [10]. The SNP profiling of the *ERCC1* mostly indicated clinical relevance for response to platinum-based CT and association with survival [12,43–46] but its association with clinical outcomes remains unclear. Several studies also evaluated the role of *ERCC2* SNPs rs13181 and rs1799793 in the prognosis and survival outcomes of advanced NSCLC. Generally, and in accordance with our results, variant genotypes were reported to be associated with decreased OS in these patients [13,44,47–49]. Interestingly, two meta-analyses reported that SNPs in both *ERCC1* and *ERCC2* genes may play a significant role in lung cancer risk. In detail, Xu et al. indicated that individuals carrying at least one wild-type allele in *ERCC1* rs11615 have a reduced risk of lung cancer development [12], while a large meta-analysis by Zhan et al., including 22 studies about *ERCC2* rs13181 and rs1799793 polymorphisms suggested that mutant genotypes of these SNPs are correlated with lung cancer development [50]. Taken together, these data highlight the dual role of DNA repair genes, both in carcinogenesis and in the restoration of platinum-induced DNA damage. For that reason, DNA repair systems have been described as a double-edged sword [7,51] because, on the one hand, a deficiency in DNA repair functions may increase cancer susceptibility, while, on the other hand, it may improve survival in patients already diagnosed with cancer, when treated with platinum agents.

A novelty of the present study is that the integrated analysis of the expression of *ERCC1* and *ERCC2* and their SNPs variants was able to confirm a trend for a longer PFS and a significantly better OS for patients with a favorable signature (low gene expression and no homozygous mutant variants) than those with unfavorable signature (high gene expression or presence of homozygous mutant variants of the SNPs). This finding supports the prognostic role of the NER pathway genes and suggests that an integrated analysis of both mRNA expression and gene polymorphisms in the major components of the NER pathway may identify SCLC patients with different survival outcomes.

Limitations of the present study include its retrospective design and the relatively small sample size that greatly weakens the statistical power of survival analysis. Furthermore, it is known that the DNA repair process includes a complex set of different mechanisms, and thus several genetic polymorphisms in other DNA repair pathways could influence the clinical outcome of SCLC. Finally, our study population received platinum-based CT, as combination treatment with ICI had not yet been approved for patients with ED-SCLC at the time of the design of the study. However, we consider that our research may help to identify a subgroup of patients who achieve substantial benefit from standard CT and with a better prognosis. Therefore, future large sample and prospective studies are warranted to validate the role of expression and polymorphisms in NER pathway genes on the prognosis of SCLC patients.

Finally, in the new era of chemo-immunotherapy and in a future therapeutic landscape in which new targeted therapies may also play a role in the therapeutic strategy of SCLC, the role of platinum-based CT will probably still remain relevant in maximizing the therapeutic benefit for these patients. Thus, future prospective trials may further investigate NER pathway genes and their role as prognostic factors for SCLC in a new and integrated therapeutic scenario.

## Supplementary Materials

Figure S1 Linkage disequilibrium (LD) block and haplotype frequencies for rs2296147 and rs1047768, rs1318, rs799793 and rs11615 in *ERCC1*, *ERCC2* and *ERCC5* genes. Intensity of color correlates with a higher linkage value.

## Data Availability

The datasets generated during and/or analyzed during the current study are available from the corresponding author upon reasonable request.

## Abbreviations

SCLC: Small-cell lung cancer
ED-SCLC: Extended disease-SCLC
LD-SCLC: Limited disease-SCLC
ICIs: Immune checkpoints inhibitors
CT: Chemotherapy
NER: Nucleotide excision repair
ERCC: Excision repair cross-complementing group
SNPs: Single nucleotide polymorphisms
CR: Complete response
PR: Partial response
SD: Stable disease
PD: Progression disease
RECIST: Response evaluation criteria in solid tumors
PFS: Progression-free survival
OS: Overall survival
ECOG PS: Eastern cooperative oncology group performance status
FFPE: Formalin-fixed paraffin-embedded
PCI: Prophylactic cranial irradiation
LD: Linkage disequilibrium

## Supplementary Materials

The supplementary material is available online at https://doi.org/10.32604/or.2024.050161.
