# Supplementary figures and images for: Genetic signatures of ERCC1 and ERCC2 expression, along with SNPs variants, unveil favorable prognosis in SCLC patients undergoing platinum-based chemotherapy
Source: Oncol Res. 2024 Dec 20;33(1):45–55. doi: 10.32604/or.2024.050161 (PMC11671403; doi:10.32604/or.2024.050161)

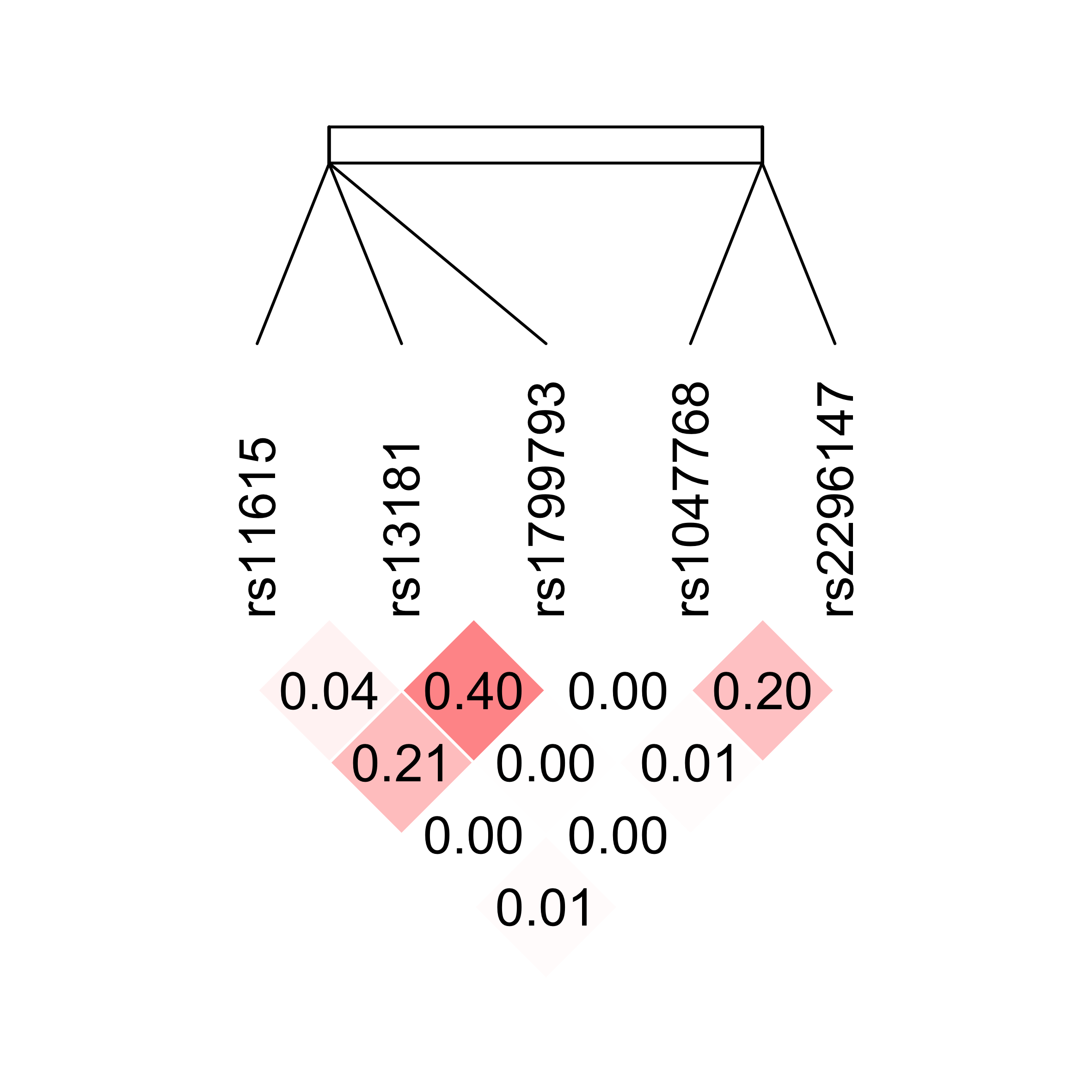

Supplement: Figure S1 [file OncolRes-33-50161-s001.tiff]
