# Supplementary material for: Genetic signatures of ERCC1 and ERCC2 expression, along with SNPs variants, unveil favorable prognosis in SCLC patients undergoing platinum-based chemotherapy
Source: Oncol Res. 2024 Dec 20;33(1):45–55. doi: 10.32604/or.2024.050161 (PMC11671403; doi:10.32604/or.2024.050161)
Supplement: Supplementary file 2 [file OncolRes-33-50161-s002.docx]

**Table S1.** Frequency of genotypes in *ERCC1*, *ERCC2* and *ERCC5* genes in the study population

| **Gene** | | *ERCC1* | *ERCC2* | | *ERCC5* | |
| --- | --- | --- | --- | --- | --- | --- |
| **SNP ID** | | rs11615 | rs13181 | rs1799793 | rs1047768 | rs2296147 |
| **Chromosome location** | | chr19:45420395 | chr19:45351661 | chr19:45364001 | chr13:102852167 | chr13:102846025 |
| **variant** | | A>G | T>G | C>T | T>C | T>C |
| **SNP type** | | synonymous variant | stop gain variant | missense variant | missense variant | intron variant |
| **genotype** | homozygous reference | A/A= 15 (39%) | T/T = 12 (32%) | C/C = 10 (26%) | T/T = 7 (18%) | T/T = 2 (5%) |
|  | heterozygous | A/G= 16 (42%) | T/G = 20 (53%) | C/T = 22 (58%) | T/C = 19 (50%) | T/C = 18 (47%) |
|  | homozygous alternative | G/G= 7 (18%) | G/G = 6 (16%) | T/T = 6 (16%) | C/C = 12 (32%) | C/C = 18 (47%) |
| **SCLC cohort alleles frequencies** | reference | 60% | 57.5% | 55% | 43% | 28.5% |
|  | alternative | 40% | 42.5% | 45% | 57% | 70.5% |
| **general population alleles frequencies** | reference | 62% | 63% | 65% | 41% | 52% |
|  | alternative | 38% | 37% | 35% | 59% | 48% |
| **χ^2^ Test** | χ^2^ | 0.07 | 0.85 | 3.17 | 0.18 | 16.2 |
|  | *p* | *0.79* | *0.36* | *0.075* | *0.67* | *<0.0001* |
