# Supplementary material for: Genetic signatures of ERCC1 and ERCC2 expression, along with SNPs variants, unveil favorable prognosis in SCLC patients undergoing platinum-based chemotherapy
Source: Oncol Res. 2024 Dec 20;33(1):45–55. doi: 10.32604/or.2024.050161 (PMC11671403; doi:10.32604/or.2024.050161)
Supplement: Supplementary file 3 [file OncolRes-33-50161-s003.docx]

**Table S2.** Progression free survival (PFS) and overall survival (OS) according to *ERCC1, ERCC2* and *ERCC5* SNPs variants

| **SNPs** | **median PFS, mo (95% CI)** | **Log-rank *p*** | **Median OS, mo (95% CI)** | **Log-rank *p*** |
| --- | --- | --- | --- | --- |
| ***ERCC1* rs11615** | | | | |
| A/A + A/G | 5.2 (4.0-7.7) | 0.24 | 6.2 (4.6-8.9) | 0.14 |
| G/G | 4.4 (2.4-NR) |  | 4.4 (2.4-NR) |  |
| ***ERCC2* rs13181** | | | | |
| T/T + T/G | 5.1 (4.4-7.2) | 0.43 | 6.2 (4.6-7.7) | 0.21 |
| G/G | 4.0 (2.4-NR) |  | 4.8 (2.4-NR) |  |
| ***ERCC2* rs1799793** | | | | |
| C/C + CT | 5.3 (4.6-7.2) | 0.26 | 6.2 (4.9-7.7) | 0.16 |
| T/T | 3.4 (2.4-NR) |  | 4.2 (2.4-NR) |  |
| ***ERCC5* rs1047768** | | | | |
| T/T + T/C | 5.1 (3.4-9.1) | 0.62 | 6.0 (4.2-10.6) | 0.99 |
| C/C | 5.2 (2.5-NR) |  | 5.2 (4.0-NR) |  |
| ***ERCC5* rs2296147** | | | | |
| T/T + T/C | 5.2 (4.0-9.4) | 0.78 | 6.0 (4.6-10.8) | 0.66 |
| C/C | 5.0 (2.5-9.4) |  | 5.6 (2.9-8.9) |  |
